# Why Bangladesh Should Maintain the Ban on e‐Cigarettes: A Commentary

**DOI:** 10.1002/hsr2.73017

**Published:** 2026-08-09

**Authors:** Md. Golam Kibria

**Affiliations:** ^1^ Department of Research Centre for Development Action (CDA) Dhaka Bangladesh

## Abstract

**Background and Aims:**

Bangladesh has made significant progress in tobacco control; however, the emergence of electronic cigarettes (widely known as e‐cigarettes) poses a new regulatory and public health challenge. This commentary examines the implications of a potential reversal of the recent e‐cigarette ban.

**Methods:**

A narrative policy analysis was conducted, drawing on national data, global evidence, and regulatory frameworks to assess the public health and system‐level implications of legalising e‐cigarettes in Bangladesh.

**Results:**

Available evidence suggests that e‐cigarette use is increasingly concentrated among young people in Bangladesh, driven by product appeal, social media influence, and perceptions of reduced harm. Bangladesh's current regulatory and enforcement capacity is insufficient to effectively regulate a legal e‐cigarette market and is vulnerable to industry interference. Consequently, lifting the existing ban could increase youth nicotine exposure and create additional regulatory challenges, including the coexistence of legal and illicit markets.

**Conclusion:**

Lifting the e‐cigarette ban in Bangladesh would be premature and potentially harmful. Policy efforts should prioritise strengthening enforcement of the current ban, enhancing regulatory capacity, and expanding youth‐focused prevention strategies.

Bangladesh has achieved notable progress in tobacco control over the past two decades through the *Smoking and Tobacco Products Usage (Control) Act 2005* and its subsequent amendments. These legal measures have strengthened smoke‐free environments, increased public awareness, and contributed to a gradual decline in combustible cigarette use [1]. Despite these achievements, the rapid emergence of electronic cigarettes (widely known as e‐cigarettes) and vaping products presents a new public health and regulatory challenge that threatens to undermine these gains.

Globally, e‐cigarette use is pervasive among adolescents and young adults. In Bangladesh, 95% of e‐cigarette users are young people, predominantly students [2], raising serious concerns about early nicotine initiation and dependence. The appeal of flavoured products, combined with aggressive digital marketing and social media influence, has fuelled curiosity and experimentation among young people [3]. This trend is further reinforced by the widespread perception that vaping is less harmful than combustible tobacco and may aid smoking cessation, although these claims remain the subject of ongoing scientific debate. In response, Bangladesh adopted a strict regulatory position on 24 December 2025 through the *Smoking and Tobacco Products Usage (Control) (Amendment) Ordinance, 2025*, which bans the production, import, sale, marketing, and use of e‐cigarettes and related products, with implementation effective from 30 December 2025 [4]. However, only 3 months later, a parliamentary special committee has initiated a review of the ordinance, including consideration of lifting the ban. Arguments in favour of relaxation include concerns about illicit trade, claims of relative harm reduction, and the potential contribution of the industry to employment and government revenue [5]. This commentary argues that such a policy reversal would be premature and potentially detrimental to public health.

Although e‐cigarettes have been promoted by some authorities as harm‐reduction tools, this claim remains contested. While some health authorities recognise a potential role for e‐cigarettes in smoking cessation under carefully regulated clinical settings, accumulating evidence indicates that they have substantial health risks and should not be considered harmless. Most e‐cigarettes contain nicotine, a highly addictive substance that can adversely affect adolescent brain development, including cognition, attention, and impulse control [6, 7]. In addition, vaping aerosols may expose users to toxicants such as heavy metals, ultrafine particles, and volatile organic compounds. Currently available evidence also indicates that the dual use of e‐cigarettes and combustible tobacco may increase health risks rather than reduce them, thereby nullifying the potential public health benefits of e‐cigarettes as harm‐reduction products [8, 9]. These concerns are particularly relevant in Bangladesh, where e‐cigarette use is concentrated among adolescents and young adults rather than established adult smokers attempting to quit. The rising uptake of vaping among young people, therefore, raises urgent concerns about nicotine addiction and the renormalisation of nicotine use in a new generation.

Internationally, regulatory approaches to e‐cigarettes vary widely. Some countries have adopted harm reduction‐oriented regulatory frameworks, while others have implemented complete bans, particularly to protect young populations [10]. Importantly, countries with limited regulatory and enforcement capacity often face greater challenges in controlling product quality, advertising, and illegal market expansion. Bangladesh's existing ban aligns with a precautionary regulatory approach adopted in several low‐ and middle‐income countries (LMICs), where youth protection and enforcement feasibility are central considerations [11]. Given the current challenges in enforcing tobacco control legislation, including illicit trade and informal sales channels, introducing a legal vaping market in Bangladesh could further strain its regulatory systems. Moreover, as a Party to the World Health Organization Framework Convention on Tobacco Control (WHO FCTC), Bangladesh has an obligation to prevent nicotine addiction and protect public health policies from commercial and industry interference [12]. Legalising e‐cigarettes without adequate regulatory infrastructure could increase industry influence, facilitate aggressive marketing practices, and expose young people to targeted advertising.

A key argument against lifting the ban is Bangladesh's limited capacity for effective regulatory enforcement. The introduction of a legal vaping market would require robust systems for product testing, age verification, taxation, marketing regulation, and surveillance of emerging products. At present, these regulatory systems are not yet sufficiently developed to effectively oversee a legal e‐cigarette market. Additionally, the expansion of online sales and informal distribution networks poses major enforcement challenges. Even in high‐income settings, regulating digital sales of vaping products has proven difficult. In Bangladesh, where e‐commerce regulation is still evolving [13], ensuring effective compliance would be substantially more complex. Weak enforcement could result in a dual market structure: a partially regulated legal market alongside a growing illicit sector, thereby undermining both public health objectives and regulatory coherence. Although maintaining the current ban is unlikely to eliminate illicit trade entirely, it can help prevent the expansion of the legal availability, commercial marketing, and retail distribution of e‐cigarettes until adequate regulatory and enforcement capacity is established. A legal market could coexist with illicit supply, creating a dual market that would require simultaneous oversight of licensed products and efforts to combat illegal sales [14]. In contrast, maintaining the current ban enables enforcement agencies to focus their efforts on preventing the importation, distribution, sale, and promotion of prohibited e‐cigarette products while strengthening border control, market surveillance, and oversight of online sales.

In conclusion, lifting the e‐cigarette ban in Bangladesh at this stage would be premature and harmful. Existing evidence shows high youth uptake, strong product appeal, and uncertain long‐term health effects, all of which heighten the risk of nicotine addiction in a new generation. Bangladesh's current ban reflects a precautionary approach consistent with its limited enforcement capacity and youth protection priorities. Policy efforts should instead prioritise strengthening enforcement, improving surveillance, and preventing youth initiation. Maintaining the existing ban, alongside strengthening enforcement and regulatory capacity, remains the appropriate precautionary strategy to safeguard public health and preserve recent gains in tobacco control.

## Author Contributions

**Md. Golam Kibria:** conceptualization, writing – original draft, writing – review and editing.

## Funding

The author has nothing to report.

## Ethics Statement

Ethical approval was not required because this commentary is based exclusively on publicly available literature and policy documents and did not involve human participants or identifiable personal data.

## Conflicts of Interest

The author declares no conflicts of interest.

## Author Responsibility Statement

Md. Golam Kibria has read and approved the final version of the manuscript, had full access to all of the information included in this commentary, and takes full responsibility for the integrity of the content and the accuracy of the analyses and interpretations presented.

## Data Availability

Data sharing is not applicable to this article as no data sets were generated or analysed during the current study.
